# Feature Contributions and Predictive Accuracy in Modeling Adolescent Daytime Sleepiness Using Machine Learning: The MeLiSA Study

**DOI:** 10.3390/brainsci14101015

**Published:** 2024-10-12

**Authors:** Mohammed A. Mamun, Jannatul Mawa Misti, Md Emran Hasan, Firoj Al-Mamun, Moneerah Mohammad ALmerab, Johurul Islam, Mohammad Muhit, David Gozal

**Affiliations:** 1 CHINTA Research Bangladesh, Dhaka 1342, Bangladesh; mamunphi46@gmail.com (M.A.M.); misti.jannatulmawa@gmail.com (J.M.M.); writetoemran@gmail.com (M.E.H.); firojphiju@gmail.com (F.A.-M.); 2 Department of Public Health, University of South Asia, Dhaka 1348, Bangladesh; johurul@hotmail.com (J.I.); mmuhit@hotmail.com (M.M.); 3 Department of Public Health & Informatics, Jahangirnagar University, Dhaka 1342, Bangladesh; 4 School of Computer Science and Engineering, Nanjing University of Science and Technology, Nanjing 210094, China; 5 Department of Psychology, College of Education and Human Development, Princess Nourah bint Abdulrahman University, P.O. Box 84428, Riyadh 11671, Saudi Arabia; mmalmreab@pnu.edu.sa; 6 CSF Global, Dhaka 1213, Bangladesh; 7 Office of The Dean and Department of Pediatrics, Joan C. Edwards School of Medicine, Marshall University, 1600 Medical Center Dr, Huntington, WV 25701, USA

**Keywords:** daytime sleepiness, machine learning, predictive modeling, CatBoost, feature importance, adolescents

## Abstract

**Background:** Excessive daytime sleepiness (EDS) among adolescents poses significant risks to academic performance, mental health, and overall well-being. This study examines the prevalence and risk factors of EDS in adolescents in Bangladesh and utilizes machine learning approaches to predict the risk of EDS. **Methods:** A cross-sectional study was conducted among 1496 adolescents using a structured questionnaire. Data were collected through a two-stage stratified cluster sampling method. Chi-square tests and logistic regression analyses were performed using SPSS. Machine learning models, including Categorical Boosting (CatBoost), Extreme Gradient Boosting (XGBoost), Support Vector Machine (SVM), Random Forest (RF), K-Nearest Neighbors (KNN), and Gradient Boosting Machine (GBM), were employed to identify and predict EDS risk factors using Python and Google Colab. **Results:** The prevalence of EDS in the cohort was 11.6%. SHAP values from the CatBoost model identified self-rated health status, gender, and depression as the most significant predictors of EDS. Among the models, GBM achieved the highest accuracy (90.15%) and precision (88.81%), while CatBoost had comparable accuracy (89.48%) and the lowest log loss (0.25). ROC-AUC analysis showed that CatBoost and GBM performed robustly in distinguishing between EDS and non-EDS cases, with AUC scores of 0.86. Both models demonstrated the superior predictive performance for EDS compared to others. **Conclusions:** The study emphasizes the role of health and demographic factors in predicting EDS among adolescents in Bangladesh. Machine learning techniques offer valuable insights into the relative contribution of these factors, and can guide targeted interventions. Future research should include longitudinal and interventional studies in diverse settings to improve generalizability and develop effective strategies for managing EDS among adolescents.

## 1. Introduction

Excessive daytime sleepiness (EDS) among adolescents is a significant concern due to its broad and potentially adverse impact on academic performance, social interactions, emotional well-being, accident risks, and physical health. EDS, denoted as the presence of an overwhelming urge to sleep during the day, can lead to reduced cognitive performance, a diminished attention span, and lower academic achievement, as sleep-deprived students often struggle with their attention span, concentration, and information retention [1]. Socially, EDS can cause irritability, aggressiveness, or withdrawal, leading to isolation and a decreased quality of life [2]. Physically, it can contribute to chronic fatigue, metabolic deregulation with increased food consumption, obesity, and cardiovascular problems [3]. Emotionally, EDS is linked to mood disturbances such as anxiety and depression, further complicating normal adolescent emotional development [4]. Studies have highlighted the strong associations between EDS, insomnia, and depressive symptoms [5,6], emphasizing the need for a comprehensive approach to address these issues.

The prevalence of EDS among adolescents is relatively elevated, even if it varies across different populations and settings, reflecting the global importance and ubiquity of this issue [4,7]. Some of the variance in EDS among adolescents seems to reflect diverse demographic and environmental factors, as illustrated by reports showing different EDS prevalences in the same country. For example, in a Brazilian study involving 1132 adolescents, EDS was reported in 54.2% of the sample, with a higher incidence observed among females (64.3%) compared to males (35.7%) [8]. Similarly, another Brazilian study reported that 46.8% of 876 adolescents experienced EDS [9]. In South Korea, a study of 3871 high school students revealed a prevalence rate of 15.9% [10], while another study conducted by Choi et al. [11] reported an EDS prevalence rate of 11.2%, with a notable increase among those adolescents identified as internet-addicted.

Efforts to identify specific risk factors associated with EDS in teenagers have also yielded variable findings. In China, perceived social support and a higher household income were found as act as protective factors against EDS [12]. Kim et al. [13] in South Korea and Marco et al. [14] in the USA similarly found that parental employment, educational background, and socioeconomic status significantly influence sleep patterns and EDS risk. Joo et al. [10] emphasized the link between sleep disorders and EDS in South Korea, underscoring the need for improved sleep hygiene and behavioral interventions. Among Brazilian adolescents, Malheiros et al. [9] found that physical activity is protective against EDS, while high levels of the consumption of processed foods and increased screen time, particularly through social media, were associated with increased EDS rates. Similarly, Alves et al. [15] reported that EDS is prevalent among students with an insufficient sleep duration due to early school start times and the extensive use of electronic devices before bedtime. The impact of digital technology on sleep remains a major concern, with research indicating that non-academic screen time is linked to a shorter sleep duration and later bedtimes [16]. Emerging evidence suggests that problematic digital technology use contributes to poor sleep quality even after accounting for genetic and familial factors, indicating a potentially causal relationship that requires further exploration [17]. A systematic review by Brautsch et al. [18] consistently found that digital media use among older adolescents and young adults is associated with a shorter sleep duration, poorer sleep quality, and increased daytime tiredness, highlighting the need for more research to fully understand these dynamics.

An increased understanding of the risk factors and the prevalence of EDS in Bangladesh is particularly lacking, and research on adolescent sleep patterns has yet to be conducted. Further insights into inadequate sleep, (i.e., insufficient sleep duration and circadian misalignment) are likely prevalent among Bangladeshi adolescents and, based on studies elsewhere, could be linked to poor academic performance and health issues [4,7]. Applying machine learning techniques to large datasets focused on this topic may offer valuable insights into EDS by analyzing complex patterns in sleep, lifestyle, and mental health data, providing predictive models for targeted interventions. The present study aimed to investigate the prevalence and risk factors of EDS among adolescents in Bangladesh and utilize machine learning techniques to predict the risk of EDS. By addressing the specific needs of this population and employing advanced analytical methods, the study seeks to fill existing gaps in the literature and provide actionable recommendations for improving adolescent sleep health in Bangladesh.

## 2. Materials and Methods

### 2.1. Study Context and Location

This research is part of the “Mental Health Problems and Literacy, Lifestyle, and Substance Use among Adolescents” (MeLiSA) Study. The study aimed to assess mental health issues and lifestyle-related factors among high school students. It was conducted in November 2022 in Shahzadpur Upazila, Sirajganj district, one of the 64 districts in Bangladesh. Shahzadpur was selected due to its logistical support and alignment with the study’s budgetary constraints. The Upazila comprises 42 schools, with 16 situated in urban areas and 26 in rural settings. The selection of this location was based on the availability of resources, ease of access, and the ability to conduct the study within the allocated budget.

### 2.2. Sampling Strategies

A two-stage stratified cluster sampling method was employed to recruit participants for the study. Stage 1: School Stratification and Selection—Schools were first stratified based on their geographic location (urban vs. rural). Then, a random selection process was used to identify a total of seven schools as the primary sampling units. These included three schools from urban areas and four from rural regions. Stage 2: Grade Selection Within Schools—Within each selected school, a random selection was conducted to choose three specific grades (7, 8, and 9). Then, all students enrolled in these selected grades were invited to participate in the study, ensuring a representative sample from both urban and rural areas.

### 2.3. Sample Size Calculation

The sample size was calculated using an estimated prevalence of any mental disorder, 13.6%, from the National Mental Health Survey in Bangladesh [19], with a 5% margin of error and a 95% confidence interval. Accounting for urban–rural differences and a 10% non-response rate, the required sample size was 805. However, the study ultimately included 1496 participants, exceeding the calculated requirement.

### 2.4. Data Collection Process

Prior to the main study, a pilot study was conducted with 30 students to test the understandability and readability of the questionnaire. Based on the feedback from this pilot, problematic items were identified and subsequently revised by the research team during focus group discussions. The data collected during the pilot study were excluded from the formal analysis to maintain the integrity of the main study data. However, upon obtaining the necessary permissions and consents, the survey was administered in classrooms. The research team was present during the data collection process to assist students and address any queries or concerns. This approach aimed to ensure that students understood the questions and responded accurately.

### 2.5. Inclusion and Exclusion Criteria

The inclusion criteria required students to be present in the classroom during the survey and to be enrolled in grades 7, 8, or 9 at the selected schools. Exclusion criteria included students who did not provide informed consent, those with disabilities that precluded participation, and those who were absent from class during the survey. In addition, participants who provided incomplete responses regarding the outcome variables were excluded from the final analysis to ensure data quality, leading to a total of 1496 subjects.

### 2.6. Measures

#### 2.6.1. Sociodemographic Factors

The study collected comprehensive sociodemographic data from participants, including their age, gender, grade level, location (urban or rural), birth order, family type (nuclear or joint), monthly family income in Bangladeshi Taka (BDT), and parental education levels. Additionally, information on the participants’ smoking history was also gathered to provide context for their lifestyle and health behaviors.

#### 2.6.2. COVID-19 Related Information

Participants’ experiences related to COVID-19 were assessed through questions regarding whether they had personally contracted the virus, if their family or friends had been infected, and whether they had experienced the loss of family or friends due to the pandemic. Responses to these questions were recorded in a binary format (yes/no), facilitating a clear and straightforward evaluation of their COVID-19 experiences.

#### 2.6.3. Strength and Difficulties Questionnaire

The Strengths and Difficulties Questionnaire (SDQ) was utilized to evaluate emotional and behavioral disorders among adolescents. The SDQ comprises five subscales: Emotional Symptoms (e.g., feelings of unhappiness or being downhearted), Conduct Problems (e.g., engaging in fights), Hyperactivity/Inattention (e.g., frequent fidgeting), Peer Relationship Problems (e.g., social isolation), and Prosocial Behavior (e.g., sensitivity to others’ feelings). Each subscale includes five items, rated as Never = 0, Somewhat True = 1, or Certainly True = 2. A total SDQ score of 20 or above is considered abnormal. The scores for the subscales are as follows: Emotion 7–10, Conduct 5–10, Hyperactivity 7–10, Peer Problems 6–10, and Prosocial Behavior 0–4 [20,21,22].

#### 2.6.4. Depression

Depressive symptoms were assessed using the Patient Health Questionnaire (PHQ-9), which consists of nine items that participants answered based on their experiences over the past two weeks [23,24]. Scores on the PHQ-9 range from 0 to 27, with higher scores indicating more severe depressive symptoms. A cutoff score of 10 or higher was used to identify significant depression, which has a sensitivity of 88% and a specificity of 88% for major depression [23]. In this study, the PHQ-9 demonstrated a Cronbach’s alpha of 0.76, indicating good internal consistency.

#### 2.6.5. Anxiety

Anxiety levels were measured with the Generalized Anxiety Disorder (GAD-7) scale, comprising seven items rated according to the participant’s experiences over the previous two weeks [25,26]. Scores range from 0 to 21, with higher scores reflecting more severe anxiety. A cutoff score of 10 or higher was employed to identify significant anxiety, which has a sensitivity of 89% and a specificity of 82% for anxiety screening [25]. The GAD-7 also showed a Cronbach’s alpha of 0.76 in this study, signifying reliable internal consistency.

#### 2.6.6. Digital Addiction

Digital addiction was assessed using the 10-item Digital Addiction Scale for Teenagers (DAST), with responses rated on a 7-point scale (1 = never to 7 = very often), yielding scores from 10 to 70 [27]. To categorize digital addiction risk, a cutoff score of 27 was chosen, which is more than one standard deviation above the mean score of 18.67 (SD = 7.88), aligning with standard statistical practices for high-risk categories. This cutoff also exceeds the 75th percentile score of 22, reinforcing its effectiveness in identifying significant digital addiction.

#### 2.6.7. Excessive Daytime Sleepiness

Excessive daytime sleepiness was evaluated using the Pediatric Daytime Sleepiness Scale, which includes eight items designed to assess various aspects of daytime sleepiness [28]. Participants rated each item on a 5-point Likert scale from 0 (never) to 4 (always). The scale includes questions like “How often do you have trouble getting out of bed in the morning?” and “Are you usually alert most of the day?” Item 3, regarding alertness, was reverse-scored to ensure consistency in the scoring direction. The total score ranged from 0 to 32, with higher scores indicating greater daytime sleepiness [28]. A cutoff score of 15 was considered for excessive daytime sleepiness [29].

### 2.7. Ethical Considerations

The study received ethical approval from the University of South Asia, Dhaka, Bangladesh. This approval ensured that the study adhered to ethical guidelines and standards for conducting research with human participants. Before initiating data collection, approval was obtained from the relevant school authorities, including school principals and class teachers, ensuring institutional support and adherence to ethical standards. Students were provided with written informed consent forms, which required review and approval by their parents or guardians. This process was designed to ensure that participation was voluntary and fully informed. Both student assent and parental consent were mandatory for participation. Students were informed that their participation in the study was entirely voluntary. They were given the option to withdraw from the study at any time during the data collection process without any penalty. Additionally, students were not provided with any compensation for their participation, aligning with ethical standards for non-coercive recruitment practices.

### 2.8. Statistical Analyses

The data were first entered and made ready for the SPSS using Microsoft Excel 2019. Categorical variables were presented with frequency percentages, while continuous variables were reported as means and standard deviations. Associations between variables and daytime sleepiness were assessed using chi-square tests. All the study variables were entered into a logistic regression model to determine the factors associated with daytime sleepiness among adolescents. All statistical tests were conducted at a significant level of *p* < 0.05 with a 95% confidence interval.

### 2.9. Machine Learning Analyses

In this study, machine learning approaches were developed using Python programming, while data analysis and model training were conducted using Google Colab. To ensure accurate model evaluation, the dataset was divided into training and testing halves in an 80:40 ratio. To assess their efficacy, a variety of machine learning models, including Decision Tree (DT), CatBoost, Extreme Gradient Boosting (XGBoost), Random Forest (RF), and K-Nearest Neighbors (KNN), were used. These models were selected due to their extensive use in predictive modeling assignments and their proven ability to handle complex, non-linear interactions in the data. These models offer an optimal balance of interpretability, accuracy, and computational ease of use, which makes them particularly well-suited for predicting substance use behaviors. Feature importance was examined using the CatBoost and XGBoost models to identify the primary predictors of the target variable. Each model’s performance was also evaluated using a broad range of parameters, including log loss, accuracy, precision, and F1 score. The performance of the model was assessed using the following metrics:

- **Accuracy:** Accuracy is the measure of the proportion of correctly predicted cases to all examples, which establishes the overall correctness of the model. It provides a summary of the general performance of the model.
- **Precision:** Also referred to as positive predictive value, precision is the proportion of correctly predicted positive outcomes among all positive predictions the model generates. It is significant because it indicates the proportion of projected positives that are truly accurate, especially in scenarios where false positives could be costly.
- **F1 score:** By calculating the harmonic mean of these two parameters, the F1 score strikes a balance between recall and precision. It is especially useful when there is an imbalance in the classes because it provides a single number that accounts for both false positives and false negatives.
- **Log loss:** Also known as logistic loss or cross-entropy loss, log loss measures the level of uncertainty in the model’s predictions by penalizing incorrect classifications based on their expected probability. It clarifies the model’s calibration and level of confidence in its probabilistic forecasts.

### 2.10. Machine Learning Models

#### 2.10.1. K-Nearest Neighbors (KNN)

Among instance-based learning strategies, K-Nearest Neighbors (KNN) is unique in that it delays computation until after classification. This non-parametric method resolves regression and classification problems using locally determined functions. KNN finds the K training samples that are closest to the item that needs to be classified in the feature space. An object’s K nearest neighbors cast a majority vote to determine its class membership; K is frequently a small positive number. When K = 1, the item is assigned to the class of its nearest neighbor [30].

#### 2.10.2. Random Forest (RF)

A well-liked ensemble learning method, Random Forest is renowned for its perseverance in tackling classification and regression issues. During training, a large number of decision trees are constructed using this method, from which the class mode (in the case of classification) or mean prediction (in the case of regression) is obtained. By bagging training models with different fractions of the training data, Random Forests reduce overfitting in decision trees and improve prediction accuracy. Consequently, a more accurate and comprehensive model is produced by the diverse forest of trees [31].

#### 2.10.3. Gradient Boosting (XGBoost)

XGBoost represents a significant breakthrough in ensemble machine learning approaches, outperforming conventional gradient-boosting methods thanks to its innovative gradient-boosting architecture. Algorithmic design and systems optimization have greatly advanced because of this technology [32]. It enables the successive building of decision trees, minimizing errors by using the mistakes made by previous trees as a guide. Because of XGBoost’s methodical approach to speed and performance enhancement and its deft handling of large-scale data, predictive modeling has evolved, surpassing the accuracy of its GBM predecessors [33].

#### 2.10.4. Categorical Boosting (CatBoost)

Classified data are a good fit for CatBoost and other modern machine learning techniques. Gradient boosting using decision trees is the main focus. Recently, Yandex created a brand-new technique called CatBoost that reduces common problems with categorical data without necessitating a large amount of pre-processing. It accomplishes this by fusing one-hot encoding with an advanced computational technique that improves prediction accuracy and decreases overfitting. CatBoost is a very useful tool for many applications, including recommendation systems and predictive modeling, due to its well-known scalability and effectiveness. The method is significant in the rapidly expanding field of machine learning and has been demonstrated to enhance model performance, especially in datasets with a high concentration of categorical variables [34].

#### 2.10.5. Gradient Boosting Machine (GBM)

Gradient Boosting Machine (GBM), a potent ensemble machine learning technology, creates a strong learner by progressively adding weak learners, usually decision trees. This increases predicted accuracy. Gradient descent is used in this method to minimize the loss function, with an emphasis on areas where prior models commonly performed badly. Although careful hyperparameter tuning is necessary to avoid overfitting and to control the computing costs, GBMs are adaptable and powerful for a variety of prediction applications. They are extensively utilized in a variety of industries due to their capacity to manage complex, nonlinear data [33].

#### 2.10.6. Support Vector Machine (SVM)

Robust supervised learning methods like Support Vector Machine (SVM) are widely used for classification problems. It splits a dataset’s various class memberships as much as possible by locating the best hyperplane. Support vector machine learning (SVM) aims to improve classification accuracy by utilizing the data points in the support vectors that are closest to the decision boundary. The kernel trick increases the efficiency and adaptability of handling nonlinear data [35].

## 3. Results

### 3.1. Characteristics of the Study Participants

The study involved 1496 adolescents, with a nearly equal gender distribution (53.9% boys and 46.1% girls). The mean age of the participants was 13.91 (±1.07 years, and primarily aged between 12 and 14 years (71.9%). Participants were from Grades 7, 8, and 9, with the largest group in Grade 8 (41.4%). The sample was almost evenly divided between urban (49.1%) and rural (50.9%) areas. Most participants were first-born children (50.2%) and came from nuclear families (80.5%). Family income varied, with 14.6% earning less than BDT 15,000 monthly. However, digital device usage was prevalent, especially television (68.0%) and smartphones (52.9%), with many having these devices in their personal rooms. Health and behavioral assessments showed that 16.9% rated their health as excellent, while 9.1% reported emotional problems, 21.7% experienced conduct disorders, 6.2% had hyperactivity, and 15.1% had peer-relationship problems. Depression was reported by 17.1% of the participants, while anxiety was reported by 7.1%, and digital addiction risk by 14.2%. About 11.6% of the participants (n = 173) experienced daytime sleepiness (Table 1).

### 3.2. Associations with Sociodemographic Factors

Gender differences were significant: 15.7% of girls compared to 8.1% of boys (χ^2^ = 21.104, *p* < 0.001). Age showed a marginal link with sleepiness (*p* = 0.055); 13.9% of older adolescents (15–17 years) reported it versus 10.4% of younger adolescents (12–14 years). Students in higher grades had a greater prevalence of sleepiness (χ^2^ = 32.494, *p* < 0.001). Urban students reported more sleepiness (19.2% vs. 4.2%) compared to rural students (χ^2^ = 82.368, *p* < 0.001). Family-related factors like birth order (χ^2^ = 4.361, *p* = 0.225) and family type (χ^2^ = 1.045, *p* = 0.307) did not show significant associations with daytime sleepiness. However, family income was significantly related to sleepiness, with those with a higher income reporting a higher prevalence of daytime sleepiness (*p* < 0.001). Sleeping in a personal room did not significantly impact daytime sleepiness (χ^2^ = 0.211, *p* = 0.646) (Table 1).

### 3.3. Associations with Digital Device Usage

Digital device usage was significantly associated with daytime sleepiness. For instance, 13.7% of television users reported sleepiness compared to 7.1% of non-users (χ^2^ = 13.742, *p* < 0.001). Similarly, 17.3% of PC/laptop users experienced daytime sleepiness, compared to 10.9% of non-users (χ^2^ = 5.618, *p* = 0.018). Adolescents using smartphones and gaming gadgets reported higher levels of sleepiness compared to their non-user peers. Similarly, the presence of digital devices in personal rooms, especially PCs/laptops and gaming gadgets, showed a strong association with increased daytime sleepiness (χ^2^ = 18.170, *p* < 0.001 and χ^2^ = 17.446, *p* < 0.001, respectively) (Table 1).

### 3.4. Associations with Health and Behavioral Factors

Health and behavioral factors were highly associated with EDS. Participants who rated their health as “bad” (34.2%) or “neutral” (19.3%) were significantly more likely to report EDS compared to those with an “excellent” health rating (4.0%) (χ^2^ = 75.172, *p* < 0.001). Emotional problems were particularly impactful, with 39.0% of those experiencing emotional difficulties reporting EDS compared to 8.8% without such issues (χ^2^ = 109.874, *p* < 0.001). Other behavioral factors, such as conduct disorder (21.8% vs. 8.7%), hyperactivity (35.5% vs. 10.0%), depression (39.5% vs. 5.8%), and anxiety (54.7% vs. 8.3%), showed strong associations with EDS, with anxiety seemingly being the most robust risk factor (χ^2^ = 207.729, *p* < 0.001) (Table 1).

### 3.5. Factors Associated with Daytime Sleepiness

A logistic regression analysis was conducted to assess the predictors of EDS in adolescents. The model demonstrated statistical significance (χ^2^ = 342.613, *p* < 0.001), indicating its effectiveness in distinguishing between adolescents with and without EDS. The model accounted for 42.9% of the variance in EDS (Nagelkerke R^2^) and correctly classified 91.2% of the EDS cases.

The findings indicated that female adolescents were almost twice as likely to experience EDS compared to males (OR = 1.963, 95% CI = 1.249–3.086). Urban adolescents had a significantly higher probability of EDS than those from rural areas (OR = 2.658, 95% CI = 1.551–4.555). Poor physical condition was associated with a greater likelihood of EDS, with those reporting ‘bad’ physical condition being over five times more likely to be affected (OR = 5.600, 95% CI = 1.655–18.943). The presence of a gaming device in the personal room also increased the likelihood of disturbed sleep (OR = 3.863, 95% CI = 1.111–13.433). Adolescents facing emotional problems were more susceptible to EDS (OR = 2.857, 95% CI = 1.657–4.927). Depression was a strong predictor of EDS, with depressed adolescents being four times more likely to experience it (OR = 4.011, 95% CI = 2.498–6.441). Anxiety also notably raised the likelihood of EDS (OR = 2.693, 95% CI = 1.436–5.050). Finally, adolescents with a digital addiction risk were more than twice as likely to experience EDS (OR = 2.273, 95% CI = 1.398–3.696) (Table 2).

### 3.6. Comparative Evaluation of Machine Learning Models

#### 3.6.1. Feature Importance

**Figure 1:**
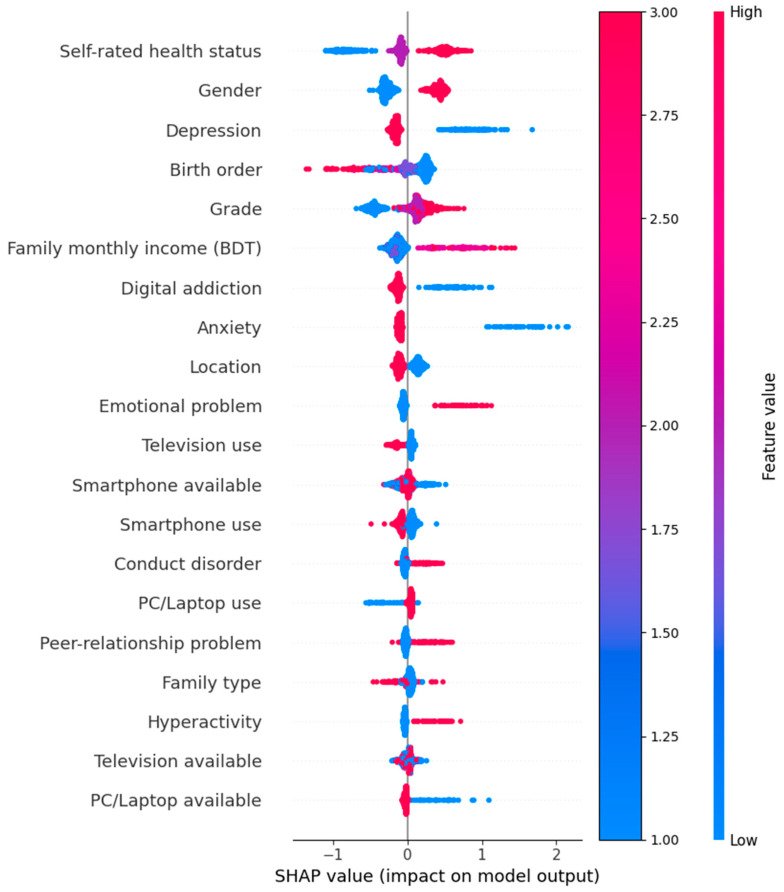
CatBoost SHAP value importance.

With the use of SHapley Additive exPlanations (SHAP) values, the impact of each feature on the distinct model predictions was measured and analyzed, offering insights into the pertinent ways in which certain characteristics influence the outcome of interest, i.e., EDS. The analysis of the SHAP values derived from the CatBoost model allows one to determine the contribution of each feature to the predictions. SHAP values offer a single, all-inclusive measure of feature significance by taking into consideration all possible interactions and determining each feature’s influence on the prediction. The analysis reveals that the top three characteristics of self-rated health status, gender, and depression have a significant impact on the model’s predictions. According to the SHAP value plot, these features have the greatest SHAP values, which suggests that they significantly affect the output (Figure 1). On the other hand, characteristics like PC/laptop available, television available, and hyperactivity were found to have the lowest SHAP values, suggesting that they have little bearing on the model’s predictions. This detailed SHAP analysis highlights how important it is to use a model-specific feature importance measure to accurately capture the nuanced contributions of each feature.

**Figure 2:**
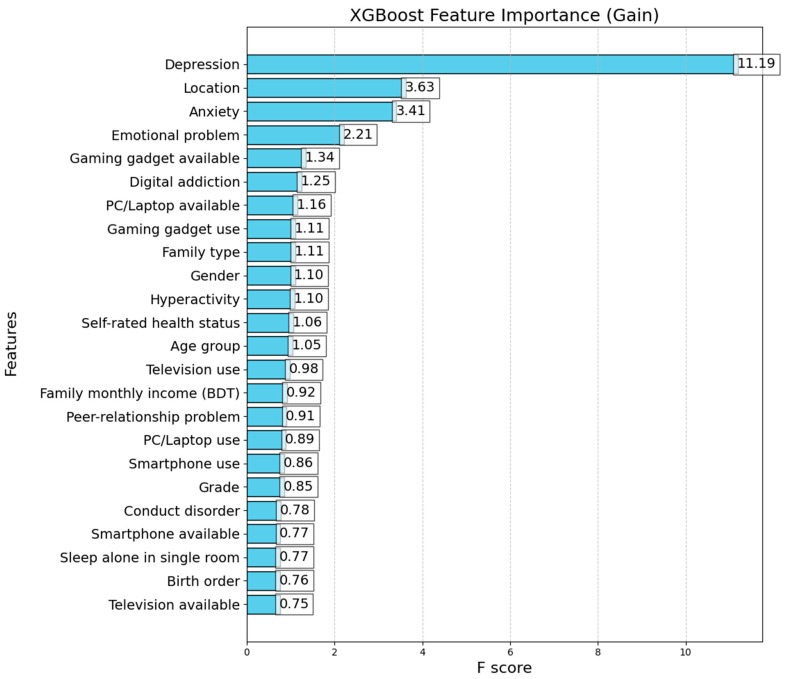
XGBoost feature importance (Gini importance).

The total impact of the decision tree-based models on each feature was assessed using Gini significance, which aided in determining the key components that affect the model’s capacity to forecast results and suggest potential areas for modification. The XGBoost model’s feature importance analysis uses Gini importance to assess each feature’s impact on the model’s prediction performance. The Gini significance, also known as Gain, quantifies the total contribution of each feature to the improvement in the model’s performance. The study shows that two of the most significant components are depression and location. These features have Gain values of 11.19 and 3.63, respectively, and have a significant impact on the model (Figure 2). The gain values of 3.41, 2.21, and 1.34 for the features of anxiety, emotional problems, and gaming gadget available indicate these features’ high significance. Television available and birth order have somewhat lower feature values (0.75 and 0.76, respectively), indicating that they have less of an effect on the model. The feature relevance distribution shows how important each variable’s properties are to the model’s capacity to forecast results.

#### 3.6.2. Machine Learning Model Performances

**Figure 3:**
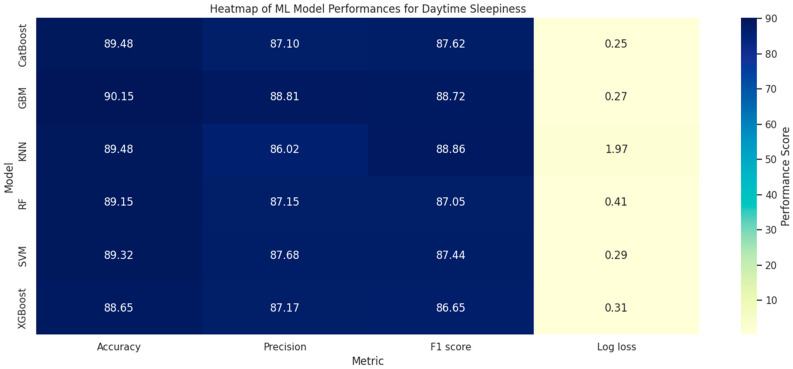
Heatmap of ML model performances for excessive daytime sleepiness.

The machine learning model’s prediction performance indicators for EDS in adolescents are shown in Figure 3. After assessments that included measures such as accuracy, precision, F1 score, and log-loss metrics, each model was found to have a differing ability to predict EDS in adolescents. Interestingly, all algorithms achieved respectable degrees of accuracy. GBM exhibited the highest accuracy score of 90.15%, CatBoost demonstrated similar accuracy of 89.48%, and XGBoost achieved the lowest accuracy of 88.65%. In terms of precision, GBM also achieved the highest score of 88.81%, while KNN achieved the lowest score 86.02%, but an F1 score of 88.86%. In comparison, XGBosst achieved the lowest value of 86.65%. Additionally, in every situation, all algorithms displayed logarithmic loss rates of less than 2%, demonstrating accurate and secure model predictions. Notably, CatBoost had the lowest log loss of 0.25 and the highest forecast accuracy, with others displaying lower scores: GBM log loss of 0.27, KNN log loss of 1.97, RF log loss of 0.41, SVM log loss of 0.29 and XGBoost log loss of 0.29. Throughout the analyses, the CatBoost model performed better than the other models in every domain, indicating its higher prediction power.

**Figure 4:**
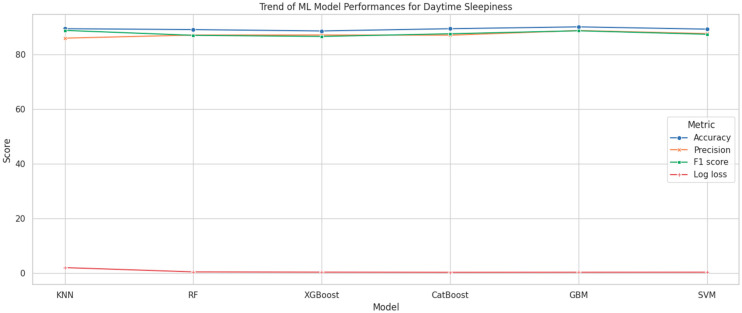
Trend of ML model performances for excessive daytime sleepiness.

Figure 4 displays the performance trends of a number of machine learning models, including XGBoost, SVM, DT, CatBoost, KNN, and RF, when evaluating a variety of factors affecting daytime sleepiness in adolescents. The models were evaluated using important metrics like Log Loss, F1 Score, Accuracy, and Precision. The results show that CatBoost outperforms the other models overall, especially in terms of accuracy and precision, with only slight modifications across multiple behaviors. The image provides a detailed comparison of the models and highlights the benefits and drawbacks of each in terms of predicting daytime sleepiness in adolescents.

#### 3.6.3. ROC-AUC Curves

**Figure 5:**
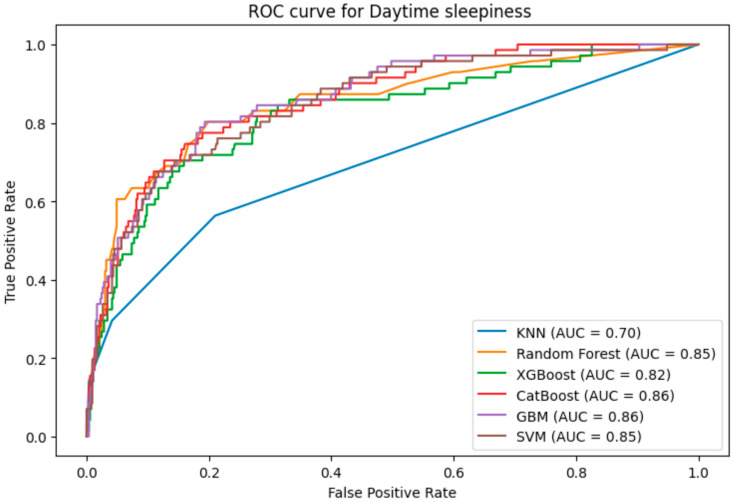
ROC-AUC curve of the model.

The algorithms’ Receiver Operating Characteristic Curve (ROC-AUC), a critical assessment parameter in machine learning for binary classification models, is shown in Figure 5. The CatBoost and GBM models showed remarkable accuracy in differentiating between positive and negative EDS classifications, with an AUC score of 0.86. RF and SVM exhibited strong discriminatory power, as evidenced by their maximum AUC score of 0.85 for EDS. Similarly, the KNN model yielded an AUC score of 0.70, which was the lowest value. Overall, CatBoost and GBM showed the strongest discriminatory power of all models based on their AUC values, effectively and competently differentiating EDS-positive and EDS-negative cases.

## 4. Discussion

This study found that the prevalence of EDS among adolescents in our study is approximately 11.6%, notably lower than some reported rates in other countries, reflecting the previously detected significant regional variability in EDS. Furthermore, an analysis of several potential risk factors enabled the construction of a predictive model that was further confirmed and strengthened using machine learning approaches, yielding high accuracy in the prediction of EDS in Bangladeshi adolescents.

Our findings are similar to those of Choi et al. [11] in South Korea, who reported an EDS prevalence of 11.2%, and to those of Joo et al. [10], who found 15.9% among South Korean high school students. In contrast, much higher EDS rates were reported in Brazil, with Meyer et al. [8] reporting rates of 54.2% and Malheiros et al. [9] reporting rates of 46.8%. These discrepancies may be influenced by demographic, cultural, and socioeconomic factors, such as sleep habits, academic pressures, and lifestyle differences. For example, Brazil’s high prevalence could be related to socioeconomic factors and lifestyle habits, whereas South Korea’s lower rates might be linked to structured daily routines and a cultural emphasis on academic achievement. These differences underscore the importance of localized research to understand the factors contributing to EDS and to tailor interventions effectively. The lower EDS rate in our sample compared to the international data emphasizes the need for region-specific studies to explore local factors affecting sleep patterns and their health implications.

The study found that female adolescents are nearly twice as likely to experience daytime sleepiness compared to their male counterparts. This gender disparity aligns with the findings from various international studies. Hormonal differences, such as those related to menstrual cycles, have been shown to impact sleep patterns and quality, potentially contributing to this gender discrepancy [36]. Furthermore, societal pressures and psychosocial stressors, which disproportionately affect females, may also contribute to their increased likelihood of experiencing EDS. In addition, this study also revealed that urban adolescents are significantly more likely to experience EDS compared to their rural counterparts, a finding that has been supported by studies conducted in other parts of the world. For instance, Joo et al. [10] noted that urban adolescents face higher levels of academic stress and environmental stressors, while another study identified urbanization as a factor contributing to poor sleep quality and increased sleep disturbances among adolescents [13]. Urban environments often involve higher levels of noise, light pollution, and more stringent and competitive academic demands, all of which can adversely affect sleep quality and contribute to daytime somnolence.

In the context of digital technology use, excessive exposure to digital media has been extensively associated with poorer sleep outcomes. Burnell et al. [16], Lund et al. [37], and Perez-Chada et al. [38] found that high levels of non-academic screen time are associated with disrupted sleep patterns and decreased sleep quality. Problematic digital technology use adversely affects sleep quality, even when accounting for genetic and environmental factors [17]. Similarly, Chung et al. [39] reported that smartphone addiction among Korean adolescents correlates with increased daytime sleepiness, underscoring the impact of digital distractions on sleep. The current study corroborated that the presence of a gaming device in adolescents’ personal bedrooms is linked to a higher likelihood of EDS. Gaming devices often lead to delayed bedtimes and increased mental stimulation, promoting arousal and disrupting sleep onset and quality. Evidence shows that screen exposure before bedtime interferes with circadian rhythms, contributing to insomnia [17]. A systematic review by Brautsch et al. [18] highlighted that digital media use among adolescents and young adults is associated with shorter sleep duration, poorer sleep quality, and increased daytime tiredness. Our results, which indicate that the risk of digital addiction more than doubles the likelihood of daytime sleepiness, reinforce the need for interventions that manage screen time and promote healthier sleep practices to counteract the negative effects of digital media on sleep.

The association between poor physical condition and a higher likelihood of EDS in this study highlights the critical role that overall health plays in sleep patterns among adolescents. This finding is consistent with research by Taheri et al. [3], who linked poor physical health with increased fatigue, contributing to sleep disturbances. Adolescents in poor physical condition often experience chronic fatigue and related health issues, which exacerbate daytime sleepiness, emphasizing the need for a comprehensive approach to managing the constellation of sleep problems. Moreover, our findings indicate that adolescents with emotional problems are significantly more likely to suffer from EDS. This aligns with the work of Liu et al. [5] and Luo et al. [6], who identified strong associations between emotional disturbances and sleep issues. Emotional problems, including stress and mood disturbances, can increase physiological arousal, impair sleep onset, and disrupt sleep maintenance. EDS is also associated with a higher risk of mood disturbances, such as anxiety and depression, further complicating adolescent emotional development [4]. The bidirectional relationships between sleep disturbances and mental health issues underscore the necessity of addressing emotional well-being as part of a strategy to manage daytime sleepiness effectively. Interventions focused on improving emotional health have been shown to enhance sleep quality, highlighting the importance of integrated approaches to managing both emotional and sleep health.

In the study, SHapley Additive exPlanations (SHAP) values were used to assess feature importance within the CatBoost model, providing a comprehensive understanding of how different variables influenced the predictions of EDS. The analysis revealed that self-rated health status, gender, and depression were the top contributors to the model predictions, highlighting their significant impact on EDS among adolescents. These features had the highest SHAP values, indicating their strong influence on the model’s output. Conversely, features such as the availability of a PC/laptop, television, and hyperactivity had the lowest SHAP values, suggesting minimal impact on the model predictions. In comparison, the Gini importance analysis from the XGBoost model identified depression and location as the most significant predictors, with Gain values of 11.19 and 3.63, respectively. Other important features included anxiety, emotional problems, and the availability of gaming gadgets, with each contributing significantly to the model’s predictive performance. Features such as television availability and birth order had lower Gini importance values, indicating their lesser impact. The distribution of feature importance across these models highlights the varying predictive power of different features depending on the algorithm being used, emphasizing the need for the careful selection and interpretation of features in machine learning models. This comprehensive approach offers deeper insights, helping to improve targeted interventions for adolescents at risk.

When evaluating the performance of different machine learning models, the study found that all algorithms demonstrated adequate and satisfactory levels of accuracy in predicting EDS among adolescents. Importantly, all models maintained low log loss values, with CatBoost achieving the lowest log loss, indicating its superior prediction accuracy and reliability. The performance of the CatBoost model consistently outshined other models across multiple metrics, suggesting its robustness in predicting outcomes related to EDS. These results highlight the importance of selecting appropriate machine learning models based on the specific characteristics of the dataset and the prediction task at hand. The superior performance of CatBoost, particularly in terms of log loss and accuracy, indicates its potential as a powerful tool for identifying adolescents at risk for EDS, and facilitating timely and targeted interventions.

When compared with the findings of Al-Mamun et al. [40], who explored sleep duration and insomnia among prospective university students using machine learning techniques, several key similarities and differences emerge. Both studies highlighted the effectiveness of CatBoost in predicting sleep-related outcomes, with Al-Mamun et al. [40] reporting accuracy rates of 61.27% and 73.46% for predicting sleep duration and insomnia, respectively. In contrast, the current study achieved higher accuracy rates, particularly with GBM and CatBoost, suggesting that these models may perform differently depending on the population and specific sleep-related outcomes being studied. Moreover, while Al-Mamun et al. [40] utilized GIS techniques to explore regional variations in sleep disturbances, the present study focused more on predictive accuracy and feature importance in machine learning models. Both studies stress the unique value of machine learning in enhancing predictive accuracy and identifying significant predictors that traditional statistical methods may overlook. However, the current study’s use of SHAP values and Gini importance provides a more detailed and interpretable understanding of the feature contributions, which can be crucial for developing targeted detection and intervention measures.

Despite the strengths of this study, several limitations should be noted. First, the cross-sectional design of the research limits the ability to infer causality, as it provides only a snapshot of the associations between variables at a single point in time. Additionally, the reliance on self-reported data introduces the potential for biases, such as social desirability or recall bias, which may affect the accuracy of the reported information. The generalizability of the findings may also be constrained by the specific demographic and geographic context of Bangladesh (e.g., selecting a single district), and caution is needed when applying these results to other populations or geographical settings. The categorization of digital device usage into broad categories may oversimplify its impact, while more detailed measures could offer a more nuanced understanding. Furthermore, while several sociodemographic and behavioral variables were controlled for, there may be additional unmeasured confounders that could influence the outcomes. The study sample, although diverse, may not fully capture the variability within the adolescent population, and future research should consider a broader range of factors. Lastly, the machine learning models used in this study, while advanced, are limited by the quality of the input data and the chosen features, which may affect the interpretation of results. Addressing these limitations in future studies will be crucial for enhancing the understanding and generalizability of findings related to EDS among adolescents.

## 5. Conclusions

In conclusion, this study provides valuable insights into the prevalence and risk factors associated with daytime sleepiness among adolescents in Bangladesh, emphasizing the significance of digital device use, sleep quality, and mental health variables. The application of machine learning techniques allowed for a robust analysis of the predictive factors, revealing important associations that contribute to the understanding of EDS in this vulnerable population. The findings emphasize the need for targeted interventions that address both digital usage and mental health to mitigate the impact of daytime sleepiness. Despite the study’s limitations, including its cross-sectional design and reliance on self-reported data, the results offer a foundation for future research and practical applications. Further studies are needed to explore these relationships longitudinally and in diverse settings to enhance generalizability and inform comprehensive strategies for managing daytime sleepiness among adolescents. Overall, this study contributes to the growing body of knowledge on adolescent health and provides a basis for future work aimed at improving well-being and functioning in this critical developmental period.

## Figures and Tables

**Table 1:** Distribution of the factors and their associations with daytime sleepiness.

Variables	Total Sample	Excessive Daytime Sleepiness		
	n; %	With EDS; n; %	χ^2^ Value	*p*-Value
Socio-demographic information				
Age group (n = 1491)				
12–14 years	1075; 71.9%	112; 10.4%	3.687	0.055
15–17 years	416; 27.8%	58; 13.9%		
Gender (n = 1496)				
Male	807; 53.9%	65; 8.1%	21.104	<0.001
Female	689; 46.1%	108; 15.7%		
Grade (n = 1455)				
Grade 7	395; 26.4%	18; 4.6%	32.494	<0.001
Grade 8	620; 41.4%	71; 11.5%		
Grade 9	440; 29.4%	75; 17.0%		
Location (n = 1496)				
Urban	734; 49.1%	141; 19.2%	82.368	<0.001
Rural	762; 50.9%	32; 4.2%		
Birth order (n = 1420)				
First	751; 50.2%	97; 12.9%	4.361	0.225
Second	402; 26.9%	47; 11.7%		
Third	144; 9.6%	13; 9.0%		
More than third	123; 8.2%	9; 7.3%		
Family type (n = 1446)				
Nuclear	1205; 80.5%	137; 11.4%	1.045	0.307
Joint	241; 16.1%	33; 13.7%		
Family monthly income (BDT) (n = 493)				
<15,000	218; 14.6%	7; 3.2%	34.095	<0.001
15,000–30,000	182; 12.2%	35; 19.2%		
>30,000	93; 6.2%	22; 23.7%		
Sleep alone in single room (n = 1496)				
Yes	771; 51.5%	92; 11.9%	0.211	0.646
No	725; 48.5%	81; 11.2%		
Digital device usage				
Television (n = 1496)				
Yes	1017; 68.0%	139; 13.7%	13.742	<0.001
No	479; 32.0%	34; 7.1%		
PC/Laptop (n = 1496)				
Yes	156; 10.4%	27; 17.3%	5.618	0.018
No	1340; 89.6%	46; 10.9%		
Smartphone (n = 1496)				
Yes	792; 52.9%	114; 14.4%	13.178	<0.001
No	704; 47.1%	59; 8.4%		
Gaming gadget (n = 1496)				
Yes	43; 2.9%	12; 27.9%	11.562	<0.001
No	1453; 97.1%	161; 11.1%		
Digital device in personal room				
Television (n = 1496)				
Yes	731; 48.9%	97; 13.3%	4.065	0.044
No	765; 51.1%	76; 9.9%		
PC/Laptop (n = 1496)				
Yes	113; 7.6%	27; 23.9%	18.170	<0.001
No	1383; 92.4%	146; 10.6%		
Smartphone (n = 1496)				
Yes	583; 39.0%	86; 14.8%	9.488	0.002
No	913; 61.0%	87; 9.5%		
Gaming gadget (n = 1496)				
Yes	27; 1.8%	10; 37.0%	17.446	<0.001
No	1469; 98.2%	163; 11.1%		
Health and behavioral variables				
Self-rated health status (n = 1470)				
Excellent	253; 16.9%	10; 4.0%	75.172	<0.001
Good	657; 43.9%	48; 7.3%		
Neutral	522; 34.9%	101; 19.3%		
Bad	38; 2.5%	13; 34.2%		
Emotional problems (n = 1496)				
Yes	136; 9.1%	53; 39.0%	109.874	<0.001
No	1360; 90.9%	120; 8.8%		
Conduct disorder (n = 1496)				
Yes	325; 21.7%	71; 21.8%	42.921	<0.001
No	1171; 78.3%	102; 8.7%		
Hyperactivity (n = 1496)				
Yes	93; 6.2%	33; 35.5%	55.479	<0.001
No	1403; 93.8%	140; 10.0%		
Peer-relationship problems (n = 1496)				
Yes	226; 15.1%	30; 13.3%	0.761	0.383
No	1270; 84.9%	143; 11.3%		
Depression (n = 1496)				
Yes	256; 17.1%	101; 39.5%	234.894	<0.001
No	1240; 82.9%	72; 5.8%		
Anxiety (n = 1496)				
Yes	106; 7.1%	58; 54.7%	207.729	<0.001
No	1390; 92.9%	115; 8.3%		
Digital addiction risk (n = 1496)				
Yes	213; 14.2%	71; 33.3%	115.087	<0.001
No	1283; 85.8%	102; 8.0%		

**Table 2:** Results of logistic regression analysis on factors affecting daytime sleepiness in adolescents.

Variable	B	S.E.	Wald	df	Sig.	Exp(B)	95% C.I. for Exp(B) (Lower–Upper)
**15 to 17 Years Age group** (Ref: 12 to 14 years)	−0.088	0.257	0.118	1	0.731	0.915	0.553–1.515
**Female Gender** (Ref: Male)	0.674	0.231	8.539	1	0.003	1.963	1.249–3.086
**Urban Location** (Ref: Rural)	0.978	0.275	12.656	1	0.000	2.658	1.551–4.555
**Grade 8** (Ref: Grade 7)	0.223	0.321	0.484	1	0.487	1.250	0.667–2.344
**Grade 9** (Ref: Grade 7)	0.674	0.351	3.684	1	0.055	1.962	0.986–3.903
**Joint Family type** (Ref: Nuclear)	−0.045	0.274	0.027	1	0.871	0.956	0.559–1.635
**Sleep alone in single room** (Ref: No)	0.165	0.225	0.533	1	0.465	1.179	0.758–1.834
**TV use** (Ref: No)	0.217	0.285	0.583	1	0.445	1.243	0.711–2.171
**PC/Laptop use** (Ref: No)	-0.485	0.383	1.604	1	0.205	0.616	0.290–1.304
**Smartphone use** (Ref: No)	0.244	0.266	0.840	1	0.360	1.276	0.757–2.151
**Gaming gadget use** (Ref: No)	0.273	0.544	0.252	1	0.615	1.314	0.452–3.817
**TV available in personal room** (Ref: No)	0.201	0.242	0.690	1	0.406	1.222	0.761–1.962
**PC/Laptop available in personal room** (Ref: No)	0.551	0.401	1.889	1	0.169	1.735	0.791–3.805
**Smartphone available in personal room** (Ref: No)	−0.146	0.270	0.292	1	0.589	0.864	0.509–1.467
**Gaming gadget available in personal room** (Ref: No)	1.352	0.636	4.518	1	0.034	3.863	1.111–13.433
**Good Self-rated health status** (Ref: Very Good)	1.000	0.438	5.202	1	0.023	2.718	1.151–6.417
**Neutral Self-rated health status** (Ref: Very Good)	1.353	0.433	9.782	1	0.002	3.868	1.657–9.031
**Bad Self-rated health status** (Ref: Very Good)	1.723	0.622	7.675	1	0.006	5.600	1.655–18.943
**Emotional problem** (Ref: No)	1.050	0.278	14.259	1	0.000	2.857	1.657–4.927
**Conduct disorder** (Ref: No)	0.322	0.231	1.938	1	0.164	1.379	0.877–2.169
**Hyperactivity** (Ref: No)	0.752	0.316	5.678	1	0.017	2.121	1.143–3.937
**Peer relationship problem** (Ref: No)	0.029	0.284	0.010	1	0.919	1.029	0.590–1.795
**Depression** (Ref: No)	1.389	0.242	33.050	1	0.000	4.011	2.498–6.441
**Anxiety** (Ref: No)	0.990	0.321	9.527	1	0.002	2.693	1.436–5.050
**Digital addiction risk** (Ref: No)	0.821	0.248	10.954	1	0.001	2.273	1.398–3.696
**Constant**	−6.006	0.588	104.331	1	0.000	0.002	

Reference for age group: 12 to 14; gender: male; location: rural; class: 7th grade; family type: nuclear; physical condition: very good; and NO for all other variables. B: regression coefficient; S.E.: standard error; Wald: Wald chi-square test; df: degrees of freedom; Sig.: significance level (*p*-value); Exp(B): odds ratio; 95% C.I. for Exp(B): 95% confidence interval for the odds ratio.

## Data Availability

The datasets will be made available to appropriate academic parties upon request from the corresponding author due to institutional policy.
